# Deferred consent in emergency neurocritical research: experience from two prospective cohorts

**DOI:** 10.1186/s13054-026-05838-9

**Published:** 2026-02-21

**Authors:** Susan Alcock, Benjamin Blackwood, Marco Ayroso, Jai Shankar

**Affiliations:** 1 Department of Radiology, University of Manitoba, Winnipeg, MB Canada; 2 Rady Faculty of Health Sciences, University of Manitoba, Room 807K-JBRC/715 Mc Dermot Avenue, Winnipeg, MB R3E 3P5 Canada

Informed consent is a cornerstone of ethical research, grounded in the principles of the Nuremberg Code and the Declaration of Helsinki. It safeguards autonomy by ensuring that participation is voluntary, informed, and free of coercion. However, in emergency and critical care settings, patients are often incapacitated and clinical circumstances do not allow for delays in life-saving interventions. Even brief delays in urgent care or diagnostic imaging may negatively affect outcomes. Furthermore, approaching distressed substitute decision makers (SDMs) during the acute phase of a life-threatening emergency may be inappropriate or harmful as they are coping with shock and distress. These realities create substantial barriers to conventional informed consent in emergency research.

Deferred consent allows enrollment at the point of intervention, with consent obtained later. This model preserves autonomy and respect for persons while enabling time-sensitive research to proceed. Deferred consent is ethically permissible when the research poses minimal risk, prospective consent is impracticable, and the study has the potential to generate important knowledge for future patients, consistent with established emergency research ethics guidance [1]. Deferred consent has been widely accepted in low-risk emergency studies across multiple disciplines, including stroke, myocardial infarction, trauma, and traumatic brain injury (TBI).

The primary aim of our study was to evaluate the success of using deferred consent in two prospective cohort studies involving critically ill patients, and to identify factors associated with successful deferred consent. Two prospective cohort studies (Table 1) were conducted at the University of Manitoba: *Early Diagnosis of Mortality Using Admission CT Perfusion in Severe Traumatic Brain Injury (ACT-TBI)* [2, 3] and *CT Perfusion for Assessment of Poor Neurological Outcome in Comatose Cardiac Arrest Patients (CANCCAP*) [4, 5]. Both studies evaluated the diagnostic accuracy of CT perfusion (CTP) in predicting early mortality, in distinct populations, severe TBI patients and out of hospital cardiac arrest patients. Both studies were conducted in an urban Canadian setting (a provincial trauma center for ACT-TBI and a provincial cardiac center for CANCCAP). Participants in both studies were being actively resuscitated and incapacitated at the time of hospital arrival and thus prospective consent was not feasible. Enrollment occurred at the time of CTP acquisition, which was performed shortly after hospital admission without interrupting clinical care. To ensure methodological validity, the research CTP scan had to be performed as early as possible following hospital arrival. Although CTP provided no direct therapeutic benefit, it required immediate performance to accurately evaluate its potential as a prognostic tool. Immediate CTP is not standard of care, and delaying imaging to obtain consent would compromise data validity. The anticipated population-level benefit supported the ethical appropriateness of deferred consent despite minimal radiocontrast risk.

**Table 1:** Demographics and clinical characteristics of patients approached in ACT-TBI and CANCCAP

	Total (*n* = 286)	ACT-TBI (*n* = 196)	CANCCAP (*n* = 90)	*p*
**Age**,** median (IQR1-3)**,** y**	48 (32–64)	38 (27–55)	63 (55–71)	< 0.001
**Sex**, ***n*** **(%)**				0.539
Male	239 (83.6)	162 (82.7)	77 (85.6)	
Female	47 (16.4)	34 (17.3)	13 (14.4)	
**Presented as “Unknown”**^**a**^, ***n*** **(%)**	54 (18.9)	54 (27.6)	0 (0)	
**Status at hospital discharge**, ***n*** **(%)**				0.002
Alive	189 (66.1)	141 (71.9)	48 (53.3)	
Dead	97 (33.9)	55 (28.1)	42 (46.7)	
**Mechanism of injury**, ***n*** **(%)**				
Cardiac arrest	91 (31.8)	1 (0.5)	90 (100)	
Assault	49 (17.1)	49 (25.0)	0	
MVC	49 (17.1)	49 (25.0)	0	
ATV accident	11 (3.8)	11 (5.6)	0	
Snowmobile accident	3 (1.0)	3 (1.5)	0	
Substance abuse	17 (5.9)	17 (8.7)	0	
Fall	38 (13.3)	38 (19.4)	0	
GSW	9 (3.1)	9 (4.6)	0	
Seizure	5 (1.7)	5 (2.6)	0	
Hanging	2 (0.7)	2 (1.0)	0	
Other	4 (1.4)	4 (2.0)	0	
Unknown	8 (2.8)	8 (4.1)	0	
**Type of consent obtained**, ***n*** **(%)**				
Deferred	252 (88.1)	164 (83.7)	88 (97.8)	< 0.001
Waiver	34 (11.9)	32 (16.3)	2 (2.2)	< 0.001
**Who provided deferred consent**, ***n*** **(%)**				< 0.001
Patient	51/252 (20.2)	48/164 (29.3)	3/88 (3.4)	
Non-patient	201/252 (79.8)	116/164 (70.7)	85/88 (96.6)	
**Details of non-patient consent**, ***n*** **(%)**				
Spouse/partner	72/201 (35.8)	21/116 (18.1)	51/85 (60.0)	
Parent	46/201 (22.9)	46/116 (39.7)	0/85	
Sibling	22/201 (10.9)	16/116 (13.8)	6/85 (7.1)	
Adult children	41/201 (20.4)	21/116 (18.1)	20/85 (23.5)	
Other	20/201 (10.0)	12/116 (10.3)	8/85 (9.4)	
**Length of time to consent**, ***n*** **(%)**				0.035
Within 7 days Between 8–14 days Between 15–30 days Greater than 30 days	190/252 (75.4) 38/252 (15.1) 14/252 (5.6) 10/252 (4.0)	129/164 (78.7) 19/164 (11.6) 7/164 (4.3) 9/164 (5.5)	61/88 (69.3) 19/88 (21.6) 7/88 (8.0) 1/88 (1.1)	

ACT-TBI = Early diagnosis of mortality using Admission CT perfusion in severe Traumatic Brain Injury patients; ATV = all-terrain vehicle; CANCCAP = CT perfusion (CTP) for Assessment of poor Neurological outcome in Comatose Cardiac Arrest Patients (CANCCAP)-a prospective cohort study; MVC = motor vehicle collision; GSW = gunshot wound; ^a^Name of patient was not known upon emergency department arrival

Deferred consent was obtained within seven days from the patient, if capacity was regained, or from an SDM. Substitute decision-maker consent was not obtained at the bedside, even when SDMs were physically present, because seeking consent prior to imaging would have introduced delays incompatible with the immediate acquisition required for valid prognostic assessment. If repeated attempts to contact the SDM failed, a waiver of consent was sought from the institutional ethics board.

Of 291 eligible participants, 286 were included in the analysis (median age 48 years, 83.6% male). Deferred consent was obtained for 252 cases (86.6%), a waiver of consent was granted for 34 (11.9%), and five families (1.7%) refused participation. The most frequent mechanisms of injury were cardiac arrest (31.8%), assault, and motor vehicle collisions (each 17.1%). Nearly one-fifth of participants arrived unidentified, and in-hospital mortality was 33.9%.

Spouses/partners provided most consents (35.8%), followed by parents, adult children, and siblings. In a minority of cases, extended family and non-family contacts provided consent, reflecting complex social circumstances common among critically ill patients.

Multivariable analysis (Table 2) demonstrated that participation in the CANCCAP study, increasing age, and survival to hospital discharge were independently associated with higher odds of obtaining deferred consent. CANCCAP participants were significantly older (median 63 vs. 38 years, *p* < 0.001) and more often accompanied by family, which likely facilitated contact and consent. Deferred consent was obtained for 97.8% of CANCCAP participants and 83.7% of ACT-TBI participants. Conversely, waivers of consent were more frequent in ACT-TBI (16.3%) than in CANCCAP (2.2%), largely because many trauma patients lacked reachable SDMs or had outdated contact information. Most deferred consents (75.4%) were obtained within seven days, while 15.2% between eight and 14 days, and 9.6% after 15 days. Delays were attributable to patient instability, challenges contacting SDMs, or death before consent discussions could occur.

**Table 2:** Logistic regression analysis of risk factors associated with successful deferred consent

	Univariate			Multivariate		
	OR	95% CI	*P*-value	OR	95% CI	*P*-value
**CANCCAP**	8.59	2.52–53.76	0.004	5.72	1.48–37.94	0.027
**Age**,** scaled by 10 years**	1.47	1.20–1.86	< 0.001	1.38	1.10–1.79	0.009
**Female**	2.19	0.74–9.39	0.212	1.99	0.64–8.79	0.289
**Alive at hospital discharge**	1.43	0.67–2.94	0.343	2.79	1.21–6.40	0.015

OR- Odds Ratio; CI- Confidence interval; CANCCAP- CTP for Assessment of poor Neurological outcome in Comatose Cardiac Arrest Patients

Our findings demonstrate that deferred consent is feasible and effective in emergency neurocritical research. The overall consent rate of 86.6% aligns with prior literature, including Harron et al. [6], which reported an 84% rate in emergency trials. The high acceptance rate in our studies likely reflects both the minimal perceived risk of CTP imaging, and the societal values families place on research aimed at improving outcomes in critical illness.

The emotional distress experienced by SDMs can hinder early communication. Approaching families too soon may exacerbate stress and reduce willingness to participate. Allowing SDMs time to process the situation likely contributed to the high acceptance rate observed.

Although most consents in our studies were obtained within a week, investigators frequently observed that this window was emotionally challenging for families. Extending the consent window may support more comfortable and informed decision-making.

Differences between the two urban study populations highlight important social and logistical considerations. Severe TBI patients were typically younger, often victims of trauma or assault, and more frequently presented alone or with limited contact information, reflecting unstable living circumstances in some cases. In contrast, cardiac arrest patients were generally older and more often accompanied by family, simplifying communication and follow up. These differences underscore the need for flexible consent procedures tailored to patient populations.

This study is limited by its single-center context and lack of patient or SDM perspectives; however, evaluating two distinct critically ill populations provides important insight into the practical application of deferred consent.

Overall, deferred consent supports ethical and feasible research in life-threatening conditions by balancing respect for autonomy with the operational realities of emergency care.

## Data Availability

No datasets were generated or analysed during the current study.

## Abbreviations

ACT-TBI: Early diagnosis of mortality using admission CT perfusion in severe traumatic brain injury patients
CANCCAP: CT perfusion for Assessment of poor Neurological outcome in Comatose Cardiac Arrest Patients
CTP: CT-perfusion
SDM: Substitute decision maker
TBI: Traumatic brain injury
